# RETRACTED: Sabir et al. DNA Based and Stimuli-Responsive Smart Nanocarrier for Diagnosis and Treatment of Cancer: Applications and Challenges. *Cancers* 2021, *13*, 3396

**DOI:** 10.3390/cancers18132119

**Published:** 2026-06-30

**Authors:** Fakhara Sabir, Mahira Zeeshan, Ushna Laraib, Mahmood Barani, Abbas Rahdar, Magali Cucchiarini, Sadanand Pandey

**Affiliations:** 1 Faculty of Pharmacy, Institute of Pharmaceutical Technology and Regulatory Affairs, University of Szeged, Eötvös u. 6, H-6720 Szeged, Hungary; fakhra.sabir@gmail.com; 2 Department of Pharmacy, Faculty of Biological Sciences, Quaid-i-Azam University, Islamabad 45320, Pakistan; mzeeshan@bs.qau.edu.pk; 3 Department of Pharmacy, College of Pharmacy, University of Sargodha, Sargodha 40100, Pakistan; ushnalaraib@yahoo.com; 4 Medical Mycology and Bacteriology Research Center, Kerman University of Medical Sciences, Kerman 76169-13555, Iran; mahmoodbarani7@gmail.com; 5 Department of Physics, Faculty of Science, University of Zabol, Zabol 538-98615, Iran; 6 Center of Experimental Orthopaedics, Saarland University Medical Center, 66421 Homburg, Germany; magali.madry@uks.eu; 7 Department of Chemistry, College of Natural Science, Yeungnam University, 280 Daehak-Ro, Gyeongsan 38541, Republic of Korea

The journal retracts the article “DNA Based and Stimuli-Responsive Smart Nanocarrier for Diagnosis and Treatment of Cancer: Applications and Challenges” [1], cited above.

Following publication, concerns were brought to the attention of the Editorial Office regarding the relevance of several references presented in this article [1].

In accordance with standard journal procedures, the Editorial Office and Editorial Board conducted an investigation that confirmed the presence of a number of inappropriate citations. Additionally, the investigation identified unacknowledged overlaps with eight other published articles [2,3,4,5,6,7,8,9], without appropriate acknowledgement or citation. Consequently, the Editorial Board has lost confidence in the reliability of the findings and decided to retract this publication [1], as per MDPI’s retraction policy (https://www.mdpi.com/ethics#_bookmark30) (19 May 2026).

This retraction was approved by the Editor-in-Chief of the journal *Cancers*.

The authors have been informed of this retraction and have indicated their disagreement with this decision.
